# SARS-CoV-2 Aerosol Transmission Indoors: A Closer Look at Viral Load, Infectivity, the Effectiveness of Preventive Measures and a Simple Approach for Practical Recommendations

**DOI:** 10.3390/ijerph19010220

**Published:** 2021-12-25

**Authors:** Martin Kriegel, Anne Hartmann, Udo Buchholz, Janna Seifried, Sigrid Baumgarte, Petra Gastmeier

**Affiliations:** 1 Hermann-Rietschel-Institut, Technical University of Berlin, 10623 Berlin, Germany; anne.hartmann@tu-berlin.de; 2 Department for Infectious Disease Epidemiology, Robert Koch Institute, 13353 Berlin, Germany; BuchholzU@rki.de (U.B.); SeifriedJ@rki.de (J.S.); 3 Local Health Authority “Hamburg-Nord”, 20249 Hamburg, Germany; sigrid.baumgarte@protonmail.com; 4 Institute for Hygiene and Environmental Medicine, Charité-University Medicine Berlin, 12203 Berlin, Germany; petra.gastmeier@charite.de

**Keywords:** airborne transmission, infection prevention, risk assessment model, simplified approaches, SARS-CoV-2

## Abstract

There is uncertainty about the viral loads of infectious individuals required to transmit COVID-19 via aerosol. In addition, there is a lack of both quantification of the influencing parameters on airborne transmission and simple-to-use models for assessing the risk of infection in practice, which furthermore quantify the influence of non-medical preventive measures. In this study, a dose–response model was adopted to analyze 25 documented outbreaks at infection rates of 4–100%. We show that infection was only possible if the viral load was higher than 10^8^ viral copies/mL. Based on mathematical simplifications of our approach to predict the probable situational attack rate (PARs) of a group of persons in a room, and valid assumptions, we provide simplified equations to calculate, among others, the maximum possible number of persons and the person-related virus-free air supply flow necessary to keep the number of newly infected persons to less than one. A comparison of different preventive measures revealed that testing contributes the most to the joint protective effect, besides wearing masks and increasing ventilation. In addition, we conclude that absolute volume flow rate or person-related volume flow rate are more intuitive parameters for evaluating ventilation for infection prevention than air exchange rate.

## 1. Introduction

The respiratory route is the main mode of transmission for the virus causing COVID-19 (SARS-CoV-2) [1,2,3,4]. The virus is transported on particles that can enter the respiratory tract. Whereas larger particles (droplets) are only able to stay in the air for a short time and just in the near field (short range; approx. 1.5 m), because they settle down quickly, smaller particles (called aerosol particles; a few µm to approximately 50 µm) are also concentrated in the near field but can also follow the air flow and therefore cause infections in the near and far field. Epidemiologically, short-range transmission (through droplets or aerosol particles) is distinguished from long-range transmission (aerosol particles) [5].

In order to perform an infection risk assessment for airborne transmission in the far field and to introduce appropriate preventive measures, it is necessary to know the amount of aerosol particles produced by an infected person during various activities, how many viruses stick to the particles and how many viruses are necessary to cause an infection. However, this information is usually available very late during the course of a pandemic, if it can be determined at all. Another well-known approach is to use retrospective analysis of infection outbreaks that are very probably due to aerosol transmission of a single patient source to determine the unknown parameters, inhaled virus copies and necessary copies to cause an infection.

### State of the Art

The so-called aerosol particles (liquid or solid particles suspended in a gas) and droplets differ by size. Particles, both aerosol particles and macroscopic droplets, can be removed from the air by two different mechanisms: (i) air change because of mechanical or natural ventilation and (ii) deposition on surfaces. In the investigation of amplifiable microorganisms transported on particles, inactivation can also be seen as a removal method from air, because after the inactivation the microorganisms are not harmful anymore. Microorganisms deposited on surfaces may, depending on the material, have longer inactivation periods, but transmission via aerosol particles is not relevant for them anymore.

The air change rate (ACH) can be calculated using Equation (1) as the ratio of the volume flow (Q) to the room volume (V).

(1) $$\mathrm{ACH}=\frac{Q}{V}$$

Typical air change rates for indoor environments range between 0.5 1/h for residential buildings and 8 1/h for occupied rooms with several attendants, such as meeting rooms in offices [6]. An air change rate of six air changes per hour is recommended to minimize the risk of infection [7]. Therefore, the air change rates advised for hospitals are used [6,8]. Nevertheless, these recommendations are not based on infection prevention, but on the CO_2_ emission of room occupants, as well as general air quality requirements or thermal loads within the room.

The deposition rate for particles depends on their size, the air velocity, the turbulence intensity of the air movement and the ratio of surface area to room volume [9,10,11]. Thatcher et al. [10] investigated the deposition of particles on surfaces depending on the room furniture and the air speed. The deposition rate increased for larger particles, higher air speed or an increased surface area of furniture. Particles between 0.55 and 8.66 µm were considered in this study.

Similar results were also found by Offerman [11], but for somewhat smaller particles (between 0.09 and 1.25 µm). Still, the deposition rate increased for larger particles. For particles between 0.3 and 5 µm, which is considered to be the most important size range for airborne particles in equilibrium state, which will stay in the air for a long time and are able to carry viruses, the deposition rate ranges between 0.1 and 0.4 1/h.

Different authors published results from the measurement of the viral load in swabs of infected persons [12,13,14,15]. The results show that, on average, a viral load of approximately 10^6^ viral copies per ml can be measured in the days before symptom onset for the wild-type and the Alpha variant. In some patients, a viral load of up to 10^12^ viral copies per ml was also found around symptom onset. The temporal dynamic of the viral load depends on the course of the infection. Shortly before or at the onset of symptoms, infected persons carry the highest viral load (peak load). For approximately 10% of the infected persons, a raised peak load of ≥10^8^ viral copies per ml was found. Within approximately 24 h, the viral load can increase by a factor of about 100. Whether a given patient is infectious can be estimated through measuring the probability to culture the virus. In [13] it is described that viral loads of 10^6^, 10^7^ and 10^8^ viral copies per ml taken from the swab have a culture probability in a lab of 20%, 50% and 75%, respectively. With the Delta variant, the situation is somewhat different. The mean viral load is around 10^8^ viral copies per ml, significantly higher viral loads were found and the viral load decreases significantly more slowly after the peak [16,17,18,19].

Particles in exhaled air are generated in the respiratory tract. The mucus is aerosolized so that the viruses contained in the mucus are distributed to the aerosol particles formed. The higher the viral load, the more particles actually carry virus. The aerosol particles disperse in the room air. People in the room inhale the aerosol particles. A direct correlation between culture viability in the laboratory via a swab and culture viability via aerosol particles cannot be drawn, so that it is also not possible to conclude that a person is infectious above a certain viral load. Still, Eyre et al. [20] found that an increased viral load increases the probability of transmission of SARS-CoV-2.

The inactivation time depends on the pathogen and therefore has to be considered separately for each virus or bacteria investigated. For SARS-CoV-2, the inactivation time was investigated experimentally by van Doremalen et al. [21], as well as Dabisch et al. [22].

Van Doremalen et al. [21] investigated the inactivation in air as well as on different surfaces and compared it to values for SARS-CoV-1. The measured half-life of SARS-CoV-2 in air of approximately 1.1–1.2 h leads to an inactivation rate of approximately 0.6 1/h. Dabisch et al. [22] measured the decay rate of SARS-CoV-2 in air under different environmental conditions (temperature, humidity and simulated sunlight). The influence of simulated sunlight and temperature were found to be much larger than the influence of humidity; however, all aspects had a significant influence on the decay rate.

In 1978, Riley et al. [23] evaluated a measles outbreak in a suburban elementary school. Based on the number of susceptible persons (S), which have been infected (D) during each stage of infection of the source patient, the risk (P) for an infection at this stage has been calculated using Equation (2). The risk of infection is defined as the percentage of infected persons from the number of pupils not already infected or vaccinated.

(2) $$P=\frac{D}{S}$$

A Poisson distribution of the risk of infection is assumed, as well as a stationary and evenly distributed concentration of the pathogens in the room air. Equation (3) shows the Poisson distribution.

(3) $$P=1-e^{-\lambda }$$

In 1955, Wells [24] defined a size called quantum as the number of emitted infectious units, where the probability to become infected is 1 − e^−1^ = 63.2%. Hence, a quantum can be seen as a combination of the amount of emitted virus-laden aerosol particles and the critical dose, which may result in an infection in 63.2% of the exposed persons. The quantum concept and Equation (3) were combined by Riley [23] to obtain Equation (4).

(4) $$P_{q}=1-e^{-I\cdot q\cdot Q_{b,\mathrm{in}}\cdot t/\left(\lambda_{\mathrm{ACH}}\cdot V\right)}$$

In Equation (4), the relevant parameters are integrated. The probability of infection rises with the number of infectious persons (I), the quanta emission rate depending on the activity (q), the pulmonary ventilation rate of exposed susceptible persons (Q_b,in_), and the duration of stay (t), but is inversely related to the air change rate (λ_ACH_) and the room volume (V).

Equation (5) can be used to calculate the individual risk of infection depending on the ratio of the number of inhaled viral copies, N, and the number of viral copies necessary to result in an infection, N_0_ [25].

(5) $$P_{\mathrm{ind}}=1-e^{-N/N_{0}}$$

The probability P in Equations (2)–(5) can be seen as the individual risk of infection P_ind_.

If it is assumed that, statistically, in a group of susceptible persons (N_Pers_) exactly this percentage of people becomes infected, we define the attack rate (AR) in a given situation as the situational predicted attack rate, PAR_S_ (see Equation (6)).

(6) $$P_{\mathrm{ind}}={\mathrm{PAR}}_{s}$$

**Figure 1:**
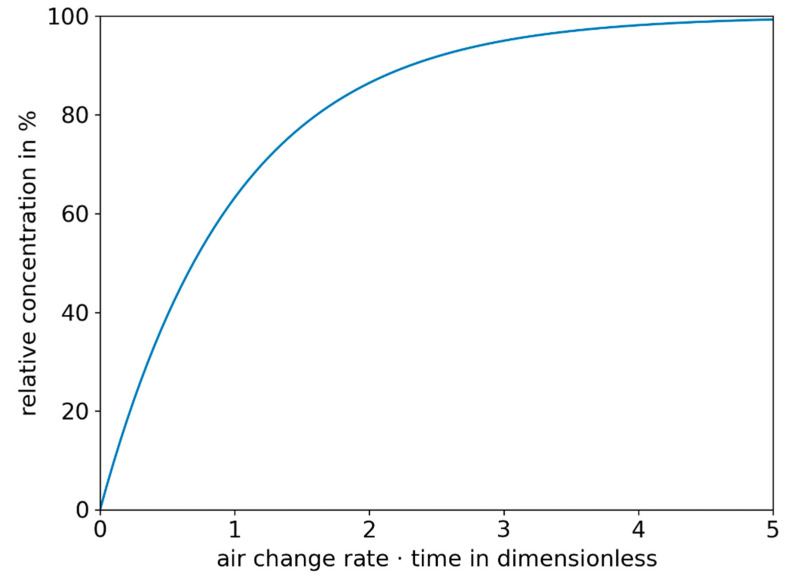
Relative concentration curve as a function of air change rate and time, based on the steady-state concentration.

In poorly ventilated rooms, the assumption of a steady concentration of virus copies is often not fulfilled. The normalized time-dependent concentration process can be calculated according to Equation (7) and is shown in Figure 1, assuming that the particles are immediately distributed uniformly in the room [26]. How rapidly the concentration of a person-emitted contamination in a room rises depends on the overall lambda (λ_g_) and the time (t). Overall lambda thereby consists of the air change rate as well as the decay rates because of sedimentation and inactivation. The relative concentration (c_rel_) based on the steady-state concentration can be seen as an increase in the concentration compared to the volume flow.

(7) $$c_{\mathrm{rel}}=1-e^{-\lambda_{g}\cdot t}$$

In many published studies that use the Wells–Riley equation to determine infection risk, ideal mixing ventilation was assumed, which means that particles are evenly distributed in the room air immediately after emission. It should be considered that local concentration differences will occur in the room and that the viral load in the room areas will vary widely [27,28,29].

To estimate the risk of infection in a given setting by a given infectious person with the Wells–Riley equation, the quanta emission rate (q) has to be known. In various studies of infection occurrences associated with SARS-CoV-2, q was determined using the Wells–Riley equation retrospectively. Different authors [30,31] found a range of 36 to 62 quanta/h with an assumed low activity (breathing, speaking) and values of 341 to 1190 quanta/h when singing. Furthermore, Buonanno et al. [32] as well as Bazant [33] used the viral load measured in the sputum of infected persons to calculate q for different activities. Therefore, emission rates for different activities (breathing volume flows, particle emission) as well as different states of infection (viral load in the sputum) can be calculated. A model applying this approach was set up by Lelieveld et al. [34].

In measurements of different research groups [35,36,37,38,39] the particle emission rates during breathing, speaking, coughing and singing were measured. During breathing through the nose, between 25 particles/s [36,38] and 135 particles/s [37] was emitted, and during coughing about 13,700 particles/cough [36], whereas it can be seen that depending on the activity a wide range of particle emission rates can be found. The particle emissions while speaking and singing depended on the loudness of the activity, but in most cases the emission rate was found to be higher for singing than for speaking. Whereas for normal speaking the emission rate ranged between 30 particles/s [38] and 270 particles/s [36,37], for singing it ranged approximately between 100 particles/s [38] and 2000 particles/s [35,40].

**Figure 2:**
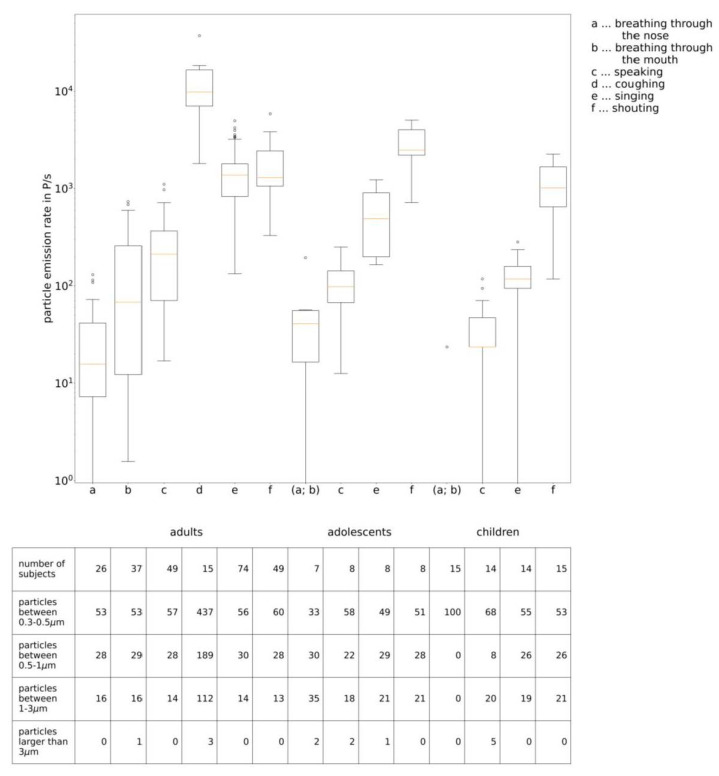
Particle emission rates measured by some of the authors, for adults [35,37,38], for adolescents [39] and for children [41].

In all five studies [35,36,37,38,40] regarding the particle emission rate of adults, at least 99% of the measured particles were smaller than 3.0 µm. In Alsved et al. [37] and Gregson et al. [38], 60% of the particles were even smaller than 1.0 µm, whereas in Hartmann et al. [36] as well as Mürbe et al. [35], 85% of the particles were smaller than 1.0 µm and 60% smaller than 0.5 µm. The particle emission rates measured by some of the authors are shown in Figure 2 and Table A1 (in the Appendix A).

Smaller particles remain airborne for a long time. The viral load (copies/mL) is aerosolized in the respiratory tract and not every small particle carries one virus. The larger the particle size, the higher the number of particles that actually carry a virus. Nevertheless, the measured particle size is the equilibrium size after evaporation. In different studies, a particle reduction between 33 and 50% [42] of the original size was measured.

Face masks can be seen as a measure to reduce the number of emitted particles, as well as the inhalation of particles from the air. The efficiency of a face mask thereby depends on three aspects:

- The filter efficiency of the fabric;
- The leakage (i.e., air flow bypassing the mask) during exhalation;
- The leakage during inhalation.

Whereas the filter efficiency of medical masks, such as surgical masks, FFP2-masks, N95 or KN95, is regulated, the efficiency of cotton masks or other homemade masks can vary widely. The results of investigations of the filter efficiency of different materials were reviewed by Kwong et al. [43]. For example, for microfiber the filter efficiency ranged from 10 to 75% and for cotton/synthetic mix from 5 to 45%. The authors conclude that the materials have to be described with more detail to make it possible to compare different studies.

Karuppasamy and Obuchowski [44], as well as Mueller et al. [45], investigated the influence of masks worn more tightly to the face. Whereas Karuppasamy and Obuchowski [44] found an improvement with surgical face masks, fixed with medical tape to the face of health care workers, Mueller et al. [45] found a reduced emission if the masks were tighter by using a nylon stocking to fix a cotton mask. The research group of Asadi et al. [46] and Cappa et al. [47] investigated the particle emission rate for different activities as well as different mask types. They found that the overall particle emission rate (through the mask as well as through leakages) was about 90% lower for coughing and 70% lower for talking compared to the case without masks. Still, this cannot be applied for cotton masks, where sometimes higher particle emission rates were measured with a mask than without a mask. In the case of a reduction, larger particles were especially found to be reduced.

In a study by some of the authors [48], the ratio of air leaking around the edges of the masks while exhaling was measured. The leakage ranged between 20 and 90% for cotton masks, between 35 and 90% for surgical masks and between 5 and 75% for FFP1 masks. The results are comparable to the results of Dreller et al. [49], who measured the leakage while inhaling.

Nevertheless, in a study of Ueki et al. [50], the influence of a mask on a mannequin emitting virus-laden particles was higher than a mask on the receiver on the amount of virus, measured as an inhalation of the receiver. The difference can be explained by the differences in airflow. Whereas for exhaling, especially large particles cannot follow the airflow and will be separated at the mask, for inhalation only smaller particles are still in the air. Therefore, the leakage might be the same, but the number of bypassing viruses is different between exhalation and inhalation, whereas masks are more helpful for the emitter than for the receiver.

Besides the number of emitted pathogen-laden aerosol particles, the number of inhaled pathogens also plays an important role with regard to the assessment of the risk of infection. The pulmonary ventilation rate may differ with different activities. Gupta et al. [51] performed a study with 25 healthy adults and found a sine wave for mere breathing, but a more constant volume flow during talking. In measurements with athletes as well as sedentary persons, a maximum volume flow for the athletes of 200 l/min (12 m^3^/h) was found by Córdova and Latasa [52]. To measure the airflow without movement restrictions, a helmet was used by Jiang et al. [53] in 32 subjects (16 males, 16 females) during speaking with different volumes, as well as during singing.

A comparison between a machine-learning-based model and measurements of respiratory rate was performed by Dumond et al. [54].

As a conclusion, the following average values can be used for adults:

- Low activity (breathing while lying): 0.45 m^3^/h [53].
- Low activity (breathing while sitting, standing or talking): 0.54 m^3^/h [53,54].
- Singing: 0.65 m^3^/h [55].
- Mid activity (physical work): 0.9 m^3^/h [54].
- Sports: 1.2 m^3^/h [52,54].

For children, the lung volume is smaller. Therefore, the respiratory rate for children aged 14 years can be assumed to be 0.45 m^3^/h for low activity (breathing while sitting, standing, talking) [56].

Antigen tests are currently widely used to detect infected individuals. The sensitivity depends on the quality of the used product and the viral load of the tested person. This was also discussed by some authors considering the quite different sensitivities of the rapid antigen tests performed by professionals (40% [57], 60.9% [58], 64.4% [59], 64.5% [58], 79.5% [60] or 85% [61]) as well as self-tests (74.4% [60] and 82.5% [61]). In a technical report of the British Department for Health and Social Care [62], the sensitivity of rapid tests depending on the viral load was given as 96% for more than 10^7^ viral copies/mL, 92% for 10^4^ to 10^7^ viral copies/mL and 43% for lower viral loads. The value for the highest viral load was also confirmed by Lindner et al. [61], but seems pretty high for the other groups compared with the values found in the aforementioned studies. In most cases, where the infected person was not detected with the rapid test, the viral load was lower than 10^6^ viral copies/mL. In [63], it was shown that suitable test kits have a sensitivity of 80% compared with RT-PCR at a viral load of 10^6^ viral copies/mL. Even the least sensitive test showed a 90% probable detection rate at a viral load of 23·10^7^ viral copies/mL. Similar orders of magnitude were found in [64]. Of 122 rapid antigen tests investigated by Scheiblauer et al. [65], 96 passed a limit of 75% sensitivity at a viral load of 10^6^ viral copies/mL. No significant change in the test sensitivity for the VOC was found [66,67]. In a model [68] as well as a longitudinal study [69], it was shown that rapid antigen tests are able to detect infected persons during the course of an infection and may therefore reduce the transmission [68] if performed at a regular frequency [69]. Whereas the viral load can increase by a factor of about 100 within 24 h before symptom onset/peak viral load [13], rapid antigen tests will detect an infection only within the diagnostic window around the highest peak of infection. It is therefore possible for an individual to receive a negative test result for a rapid antigen test despite being infected and even contagious for other persons. For this reason, an increase in regular testing frequency can greatly increase the significance of a negative test result of a rapid antigen test compared with a negative result obtained with sporadic testing. It was shown in [70] that students who had close contact with a classmate who tested positive for SARS-CoV-2 and were subsequently tested daily avoided days absent from school, with no impact on overall infection incidence.

*Based on the state of knowledge to date, the following research questions are addressed and will be answered below:*

- What viral loads are necessary to infect others via aerosol?
- Which are the most influencing factors regarding airborne transmission?
- Can a risk assessment model be simplified to allow practical recommendations?
- Is there a possibility to implement a simple measurement system for infection risk?
- What is the impact of different prevention measures on the risk of airborne transmission?

## 2. Materials and Methods

### Dose–Response Model to Predict the Individual Infection Risk and the Predicted Attack Rate (PAR)

Equations (4) and (5) assume an immediate homogeneous distribution of all emitted respiratory viruses within the room and steady-state (time independent) situations.

In the following consideration, two different cases, one stationary and one time dependent, are taken into account. For unsteady situations, it is assumed that the infected person enters the room at time 0 and the concentration in the room increases until a steady state is reached (see Figure 1).

The number of inhaled particles, N_inh_, in Equation (5) can be described with the help of Equation (8). S_V_ is thereby the viral-emission rate in viral copies/time and $\frac{S_{V}}{\lambda_{g}\cdot V_{R}}$ is the viral concentration per cubic meter of air. The overall lambda consists of the air change rate (ACH = λ_ACH_), the decay rate because of inactivation (λ_in_) and the decay rate because of sedimentation (λ_sed_).

Finally, Equation (11) can be set up.

(8) $$N_{\mathrm{inh}}=C_{V}\cdot Q_{b,\mathrm{in}}\cdot t$$

(9) $$C_{V}=\frac{S_{V}}{\lambda_{g}\cdot V_{R}}$$

(10) $$\lambda_{g}=\lambda_{\mathrm{ACH}}+\lambda_{\mathrm{in}}+\lambda_{\mathrm{sed}}$$

(11) $$P_{\mathrm{ind}}=1-e^{\left(-\frac{S_{V}}{N_{0\cdot }\lambda_{g}\cdot V_{R}}\cdot Q_{b.\mathrm{in}}\cdot t\right)}$$

If face masks are used, the number of inhaled particles N_inh_ can be reduced. This reduction can be implemented into Equation (11) as factor f_M_, which will result in Equation (12).

(12) $$P_{\mathrm{ind}}=1-e^{\left(-\frac{S_{V}}{N_{0\cdot }\lambda_{g}\cdot V_{R}}\cdot Q_{b,\mathrm{in}}\cdot t\cdot f_{M}\right)}$$

For the unsteady calculation, the course of the virus concentration is used according to Equation (7). Equation (5) therefore transforms into Equation (13) and Equation (4) into Equation (14).

(13) $$P_{\mathrm{ind}}=1-e^{-\frac{1}{N_{0}}\cdot \int C_{V}\left(t\right)\cdot Q_{b,\mathrm{in}}\cdot f_{M}}$$

(14) $$P_{q}=1-e^{-\cdot \int C_{q}\left(t\right)\cdot Q_{b,\mathrm{in}}\cdot f_{M}}$$

(15) $$C_{q}=\frac{q}{\lambda_{g}\cdot V_{R}}$$

If the individual infection risk P_ind_ approximates statistically to P_q_ (Equation (16)), Equation (17) can be obtained, where PAR_S_ i_s_ defined as the situational predicted attack rate, the attack rate during a stay in a room with infected persons.

(16) $$P_{\mathrm{ind}}=P_{q}={\mathrm{PAR}}_{S}$$

(17) $$q=\frac{S_{V}}{N_{0}}$$

To retrospectively analyze the outbreaks investigated in the following considerations, four categories of influencing factors are distinguished: first, the emission rate (viral copies per time) in connection with the critical amount of virus to result in an infection N_0_; second, the parameter C_R_, which takes the boundary conditions of the room as well as the time of stay into account; third, the breathing volume flow of the inhaling person Q_b,in_; and fourth, the total filter efficiency of the face masks considered by the filter factor f_M_.

1. Virus-related factor (VF)

The emission rate of virus-laden particles depends on the activity, which influences the number of emitted particles as well as their size distribution. Furthermore, the viral load influences the number of viruses carried on one particle. The emission rate S_V_ can therefore be described as the product of the particle emission rate N_p_, a factor considering their size distribution f_p_ and the viral load n_v_ (see Equation (18)).

(18) $$S_{V}=f_{P}\cdot N_{p}\cdot n_{V}$$

Thereby f_p_ describes a conversion factor from the particle emission rate per second to their volume emission rate per hour and depends on the size distribution. In the following calculations, a value of $f_{P}=1.1523\times 10^{8}\;\frac{\mathrm{mL}\cdot s}{P\cdot h}$ is used. The calculation of this conversion factor is displayed in the Appendix A.

N_0_ is assumed to be in the range of 100 to 300 viral copies [25]. The virus-related factor is defined in Equation (17).

2 Situation-related factor (SF)

In the situational factor the boundary conditions for the specific situation are considered. It therefore consists of the room volume V_R_, the overall lambda (λ_g_) and the time of stay (t). In a steady state it can therefore easily be derived from Equation (12) and will result in Equation (19). For an unsteady situation, the equation for the concentration (see Figure 1 and Equation (20)) has to be integrated to obtain Equation (21).

(19) $$C_{R,s}=\frac{t}{\lambda_{g}\cdot V_{R}}$$

(20) $$C=\frac{S_{v}}{N_{0}\cdot \lambda_{g}\cdot V_{R}}\cdot \left(1-e^{-\lambda_{g}t}\right)=\frac{S_{v}}{N_{0}}\cdot \frac{{\mathrm{dC}}_{R}}{\mathrm{dt}}$$

(21) $$C_{R}=\frac{1}{\lambda_{g}{}^{2}\cdot V_{R}}\cdot \left[e^{\left(-\lambda_{g}\cdot t\right)}+\lambda_{g}\cdot t-1\right]$$

3 Susceptible-person-related factor (SPF)

As mentioned in the state of the art, the breathing volume flow depends on the activity of the persons. Furthermore, it can be split up into the exhalation flow rate (Q_b,ex_) of the infected person and the inhalation flow rate (Q_b,in_) of the susceptible persons. To calculate the number of inhaled particles, just the inhaled volume flow rate (Q_b,in_) has to be considered.

4 Personal-protection-measures-related factor (PPF)

To calculate the total efficiency of a face mask, different factors for the infected person and the susceptible persons have to be considered.

Whereas the efficiency of the face mask carried by the infected person ($f_{m,e}$) is characterized by the reduction in the number of virus-laden aerosol particles introduced into the room air, the efficiency of the masks carried by the susceptible persons is characterized by their ability to reduce the number of inhaled virus-laden aerosol particles ($f_{m,\mathrm{in}}$). To take into account that these factors are different, Equation (22) considers the total efficiency as the product of the two efficiencies, whereas f_m,e_ as well as f_m,in_ is the ratio of particles going by the mask, or the difference between 1 and the ratio of particles separated by the mask.

(22) $$f_{M}=f_{m,e}\cdot f_{m,\mathrm{in}}$$

Equation (13) can therefore also be expressed as:

(23) $${\mathrm{PAR}}_{s}=1-e^{\left(-\frac{S_{V}}{N_{0}}\cdot C_{R}\cdot Q_{b,\mathrm{in}}\cdot f_{M}\right)}$$

For the calculation of PAR_s_, the following assumptions must be considered:

- The aerosol is ideally mixed in the room.
- The near field can contain a much higher virus-laden particle concentration, but it is neglected in the following.
- The air, which is introduced into the room, is free of virus-laden particles.
- A constant decay rate of deposition occurs (in this consideration $\lambda_{\mathrm{sed}}=0.2\frac{1}{h}$)
- A constant decay rate because of inactivation occurs (in this investigation $\lambda_{\mathrm{in}}=0.6\frac{1}{h}$)
- The concentration of virus-laden particles at the beginning of unsteady cases is 0 virus-laden particles/m^3^.

Twenty-five different outbreaks, either scientifically published or registered by the local health authorities, were selected. Only publications considering the time of stay, the activity of the persons and the room conditions (size as well as ventilation) are taken into consideration. In addition, the outbreaks either had to be attributed to the wild-type SARS-CoV-2 (e.g., by sequencing) or have occurred before 1 January 2021. Smaller outbreaks provided by local health authorities were included if they met the same criteria. The description of the outbreaks as well as the boundary conditions for the calculations of these situations can be found in Appendix C and in Table A5. The values were either taken from the publication or calculated from the data given in them. Some data were more secure due to better documentation, whereas other values were somewhat less certain, and the variance of the values was assumed. A normal distribution of the values characterized by the mean value and the standard deviation (given as the variance) was assumed. A Monte Carlo simulation was used to randomly combine values from within this range for the calculation of the outbreaks [71]. Therefore, a conclusion can be drawn about the reliability of the calculated values and their variance evoked by the uncertain boundary conditions. The outbreaks can be separated into different categories: choir rehearsals (4 outbreaks), outbreaks with higher physical activity (4 outbreaks), meetings with lower activity but many people (3 outbreaks), outbreaks in public transport (6 outbreaks) and smaller outbreaks, which were sometimes not scientifically published, but were investigated by local health authorities (8 outbreaks).

## 3. Results

For the investigated outbreaks and their known boundary conditions, the virus-related factor ($S_{V}/N_{0}$) was calculated retrospectively. Besides the results for the virus-related factor, the intermediate results for the factors C_R_ (steady (regarding Equation (19)) and unsteady (regarding Equation (21))), f_M_ and O_b,in_ can also be seen in Table 1.

**Figure 3:**
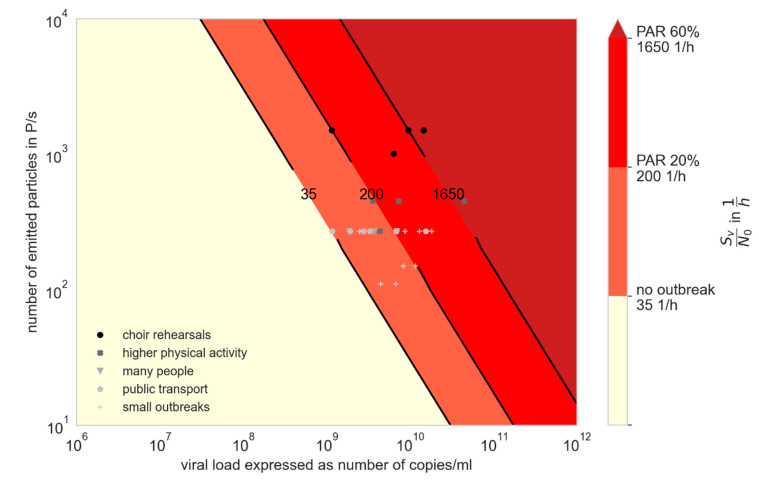
Virus factor (VF) for different viral loads and particle emission rates with N_0_ = 100 viral copies. The attack rates found in the investigated outbreaks are shown with the different colors; the markers show the amount of virus factor at assumed mean particle emission rate of the related activity according to Figure 2.

**Figure 4:**
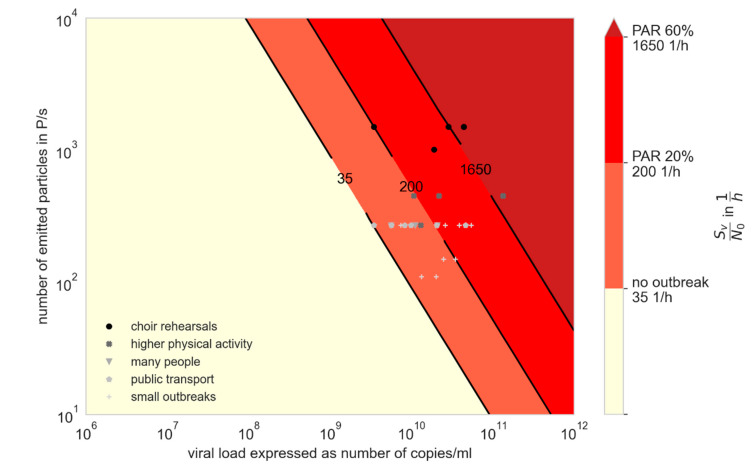
Virus factor (VF) for different viral loads and particle emission rates with N_0_ = 300 viral copies. The attack rates found in the investigated outbreaks are shown with the different colors; the markers show the amount of virus factor at assumed mean particle emission rate of the related activity according to Figure 2.

We compared the results from the retrospective investigation with available data regarding the viral load and the particle emission. Therefore, in Figure 3 and Figure 4 the ratio of the viral emission and the critical dose ($S_{V}/N_{0}$) are presented over the viral load and the particle emission. The colors visualize the AR from the investigated outbreaks, whereby the single outbreaks are shown as dots. A mean particle emission rate as a function of activity was assumed when plotting the dots, according to Figure 2. In Figure 3, the critical dose N_0_ is assumed to be the minimal value of 100 viral copies, and in Figure 4 a higher value of 300 viral copies, both related to [25]. With a higher critical dose, the lines with similar PAR_s_ are shifted upwards, whereas either a higher viral load or a higher particle emission rate is necessary to result in the same PAR_s_. It can be seen that the viral load for all investigated outbreaks had to be higher than 10^8^ viral copies/mL to explain the outbreaks with the given boundary conditions. If instead of Equation (21) for the time-dependent calculation, Equation (19) for the steady-state assumption is used, the values ${\frac{S_{v}}{N_{0}}}_{\mathrm{steady}}$ are lower than the values calculated for the unsteady conditions. Nevertheless, the viral load had to be higher than 10^8^ viral copies/mL to reach the ARs (see Table 1). 

As mentioned in Table A2, Table A3 and Table A4, some of the boundary conditions were assumed afterwards, so they are not as certain as other pieces of information. The certainty of the different boundary conditions was evaluated as quite secure (standard deviation ± 5%), somewhat insecure (standard deviation ± 20%) and insecure or unknown (standard deviation ± 50%) for each outbreak, as displayed in Table A2, Table A3 and Table A4 in the grey marked lines. To take these uncertainties into consideration, a Monte Carlo simulation with 10,000 simulations for each outbreak was performed. The investigated aspects (air change rate, room volume, number of infected persons, if it cannot be secured, that it was just one person, breathing volume flow, time of stay and attack rate) were assumed to be normally distributed with the given value as mean and the assumed level of security as standard deviation. Furthermore, limits for the AR lower than 100% and other aspects larger than 0 are considered. The median, as well as the 25% percentile and the 75% percentile, is displayed in Table 1.

In general, it can be seen that the median agrees well with the values calculated from the most probable boundary conditions. The 25% percentile is between 15% and 48% lower than the median, and the 75% percentile between 19% and 72% higher than the median. Cases with especially high deviation from the median are the Hawaiian Fitness Class and the Skagit Valley Choir, whereas most deviations ranged between 20% and 35%.

For the two choir outbreaks (Berlin 1 A and Skagit Valley C) with similar boundary conditions, quite similar ratios of S_v_/N_0_ were calculated. For lower attack rates (French Choir, D) or less singing (Berlin 2, B), the emission rate was calculated to be lower and in the same range as for the call center I, the fitness classes (F, G) or the slaughterhouse (H). Furthermore, the outbreak in the restaurant (K), the minivan 1 (P) and the club meeting (R) revealed high values for the inhaled number of infectious particles compared with the critical dose. A possible explanation is that in these situations the infectious person talked louder, because other persons talked as well or the infectious person had a higher viral emission.

For the outbreaks in schools (I, S, T, V, W, X, Y), the median values of S_v_/N_0_ ranged between 100 and 700 1/h, which is lower than for the choir or meeting outbreaks, but higher than for the other outbreaks correlated with public transport (L, M, N, O, Q).

### 3.1. Derivation of Simplified Key Figures and Calculations for the Assessment of Infection Risks and Preventive Measures

Equation (25) is created from Equation (23) with the assumption (24), where R_S_ is defined as the situational reproduction number (the number of persons probably infected during the situation), which should be statistically valid.

(24) $${\mathrm{PAR}}_{S}=\frac{R_{S}}{N_{\mathrm{Pers}}}$$

(25) $$R_{S}=N_{\mathrm{Pers}}\cdot \left[1-e^{-\frac{S_{V}}{N_{0}}\cdot C_{R}\cdot Q_{b,\mathrm{in}}\cdot f_{M}}\right]$$

This can be transformed into Equation (26).

(26) $$\ln\left(1-\frac{R_{S}}{N_{\mathrm{Pers}}}\right)=\frac{S_{V}}{N_{0}}\cdot C_{R,s}\cdot Q_{b,\mathrm{in}}\cdot f_{M}$$

For the simplified calculation, a steady-state situation is assumed. C_R,s_ can therefore be replaced by Equation (19).

(27) $$\ln\left(1-\frac{R_{S}}{N_{\mathrm{Pers}}}\right)=\frac{S_{V}}{N_{0}}\cdot \frac{t}{\lambda_{g}\cdot V_{R}}\cdot Q_{b,\mathrm{in}}\cdot f_{M}$$

In the following, it is assumed that the air change rate is the dominant variable within Equation (10). This is valid whenever non-residential standards and guidelines (DIN/ASHRAE) are respected. Therefore, $\lambda_{g}\approx \lambda_{\mathrm{ACH}}$ can be assumed. Taking Equation (28) into account, Equation (29) is obtained, with q_Pers_ as the specific volume flow (person-related volume flow) in m^3^ per hour and person.

(28) $$\lambda_{g}\cdot V_{R}=q_{\mathrm{Pers}}\cdot \left(N_{\mathrm{Pers}}+I\right)$$

(29) $$\ln\left(1-\frac{R_{S}}{N_{\mathrm{Pers}}}\right)=\frac{S_{V}}{N_{0}}\cdot \frac{t}{q_{\mathrm{Pers}}\cdot \left(N_{\mathrm{Pers}}+I\right)}\cdot Q_{b,\mathrm{in}}\cdot f_{M}$$

The following simplified method can easily be used in situations where not more than one person shall be infected, and therefore R_S_ = 1, so no outbreak would probably happen due to aerosol transmission (definition of outbreak: more than one person becoming infected during a transmission event). In rooms with a number of susceptible persons $N_{\mathrm{Pers}}\gg I$, $\ln\left(1-\frac{1}{N_{\mathrm{Pers}}}\right)\approx \frac{1}{N_{\mathrm{Pers}}}$ and $\frac{\left(N_{\mathrm{Pers}}+I\right)}{N_{\mathrm{Pers}}}≅1$ will result in a small error compared to the origin and Equation (29) can be simplified into Equation (30).

(30) $$\frac{1}{N_{\mathrm{Pers}}}=\frac{S_{V}}{N_{0}}\cdot \frac{t}{q_{\mathrm{Pers}}\cdot N_{\mathrm{Pers}}}\cdot Q_{b,\mathrm{in}}\cdot f_{M}$$

Further on, Equation (30) can be converted into Equation (31) to calculate the specific volume flow of virus-free air $q_{\mathrm{Pers}}$ to fulfill R_S_ = 1.

(31) $$q_{\mathrm{Pers}}=\frac{S_{V}}{N_{0}}\cdot t\cdot Q_{b,\mathrm{in}}\cdot f_{M}$$

In the case of a supply of virus-free outdoor air, volume flow $q_{\mathrm{Pers}}$ is correlated with CO_2_ concentration. Whereas the number of inhaled particles increases linearly with time of stay (in case of steady state), the CO_2_ concentration does not change, and caution has to be taken when using the CO_2_ concentration as an indicator for a risk of infection.

Instead of a person-related volume flow $q_{\mathrm{Pers}}$, the volume flow can also be calculated per hour of stay (see Equation (32)).

(32) $$q_{\mathrm{Pers},t}=\frac{S_{V}}{N_{0}}\cdot Q_{b,\mathrm{in}}\cdot f_{M}$$

**Figure 5:**
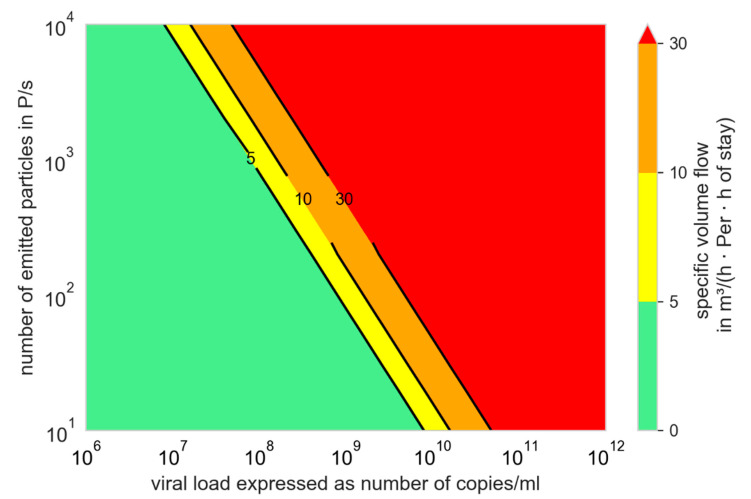
Specific volume flow depending on the number of emitted particles and the viral load to limit the number of newly infected persons to one; N_0_ = 100 viral copies, f_M_ = 1, Q_b,in_ = Q_b,ex_ = 0.54 m^3^/h.

Figure 3 can be converted into Figure 5 for Q_b,in_ = 0.54 m^3^/h (low activity (breathing while sitting, standing or talking)). In Figure 5, the specific volume flow per person and hours of stay necessary to infect not more than one further person are displayed.

The green marked area can easily be reached in most rooms for normal times of stay.

For the yellow and orange area, short times of stay or further measures have to be considered to keep the number of newly infected persons below one (R_S_ ≤ 1), whereas a much higher air supply is necessary. The volume flows in the red area cannot be reached in rooms with common airflow rates.

Instead of a specific volume flow per person q_Pers_, a specific volume per person (V_Pers_) can be used together with the overall lambda (λ_g_) to convert Equation (31) into Equation (33).

(33) $$\lambda_{g}\cdot V_{\mathrm{Pers}}=\frac{S_{V}}{N_{0}}\cdot t\cdot Q_{b,\mathrm{in}}\cdot f_{M}$$

From this equation, it can be seen that recommending an air change rate alone, e.g., 6 1/h [72,73], is not useful. In addition, the volume per person and the time of stay have to be considered, as well as virus-related properties and other preventive measures, e.g., wearing masks.

From Equation (30), Equation (34) can be derived. With this simplified approach, the maximal possible number of persons can also be found, up to which not more than one further person will become infected in the specific situation. Therefore, the available volume flow Q has to be known.

(34) $$N_{\mathrm{Pers},\max}=\frac{Q}{\frac{S_{V}}{N_{0}}\cdot Q_{b,\mathrm{in}}\cdot t\cdot f_{M}}$$

With the simplifications $\ln\left(1-{\mathrm{PAR}}_{S}\right)\approx \frac{1}{{\mathrm{PAR}}_{S}}$ and $\frac{\left(N_{\mathrm{Pers}}+I\right)}{N_{\mathrm{Pers}}}≅1$, valid for ${\mathrm{PAR}}_{S}<20\%$ and $N_{\mathrm{Pers}}\gg I$, PAR_s_ according to Equation (35) as well as R_s_ according to Equation (36) can be predicted relatively well within the limited range of values.

(35) $${\mathrm{PAR}}_{S}=\frac{S_{V}}{N_{0}}\cdot \frac{t}{q_{\mathrm{Pers}}\cdot N_{\mathrm{Pers}}}\cdot Q_{b,\mathrm{in}}\cdot f_{M}$$

(36) $$R_{S}=\frac{S_{V}}{N_{0}}\cdot \frac{t}{q_{\mathrm{Pers}}}\cdot Q_{b,\mathrm{in}}\cdot f_{M}$$

Equations (35) and (36) can be used to comparatively evaluate different situations in indoor environments as well as preventive measures. Therefore, a risk factor x_r_ can be defined according to Equation (37). If the VF remains the same in the rooms being compared (identical virus variant), then the risk factor depends on SF, SPF, and PPF only.

(37) $$x_{r}=\frac{R_{S,2}}{R_{S,1}}$$

**Figure 6:**
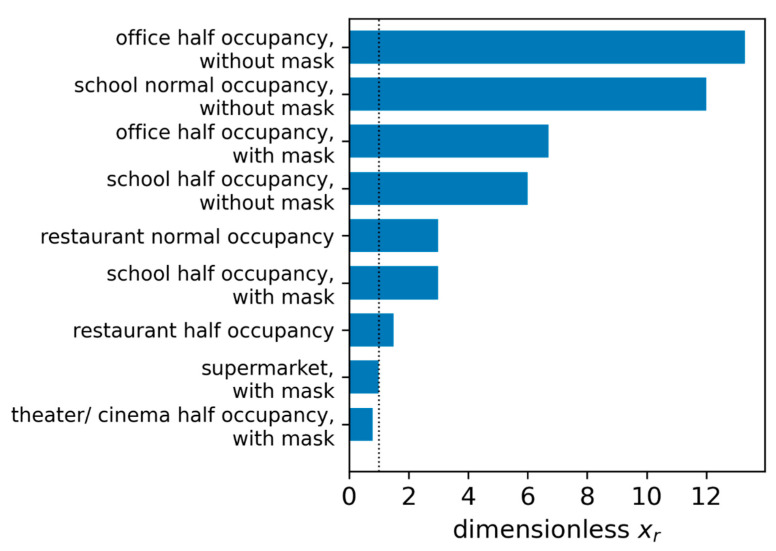
Comparison of the risk factor x_r_ for different everyday life situations with a 0.5 h stay in a supermarket, wearing a mask as reference.

In Figure 6, some everyday life situations are compared to a 0.5 h stay in a supermarket with a mask, using Equation (37). The details for these exemplary considerations can be found in the Appendix D, Table A5.

### 3.2. Influence of Variants of Concern (VOC)

**Figure 7:**
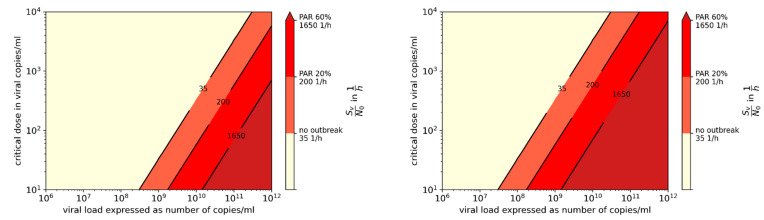
Virus factor for different viral loads and critical doses with a particle emission rate of 100 P/s, (**left**) and 1000 P/s (**right**); the attack rates found in the investigated outbreaks are shown with different colors.

For example, if the transmission rate doubles and this is not caused by a change in behavior (SF, SPF, or PPF), then it is due to the change in the VF. Here, either the necessary critical dose or the viral load, or both, may have shifted. The influence of the critical dose and S_v_/N_0_ ratio to the PAR_s_ is shown in Figure 7.

**Figure 8:**
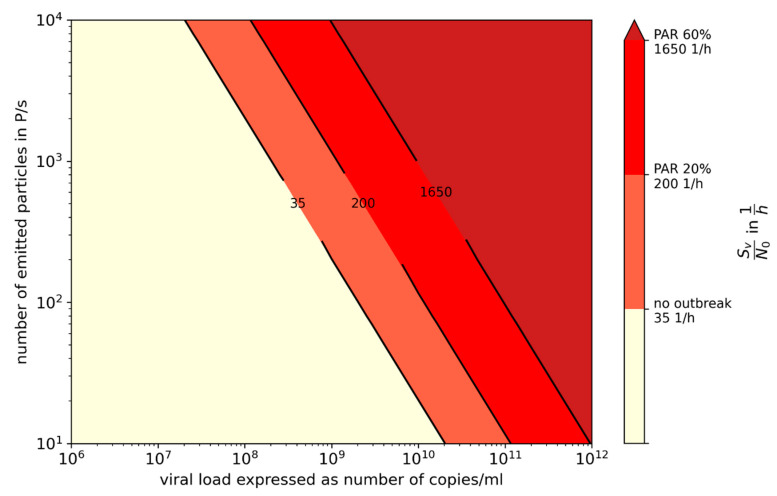
Viral emission for different viral loads and particle emission rates with N_0_ = 67 viral copies; the attack rates found in the investigated outbreaks are shown with different colors.

**Figure 9:**
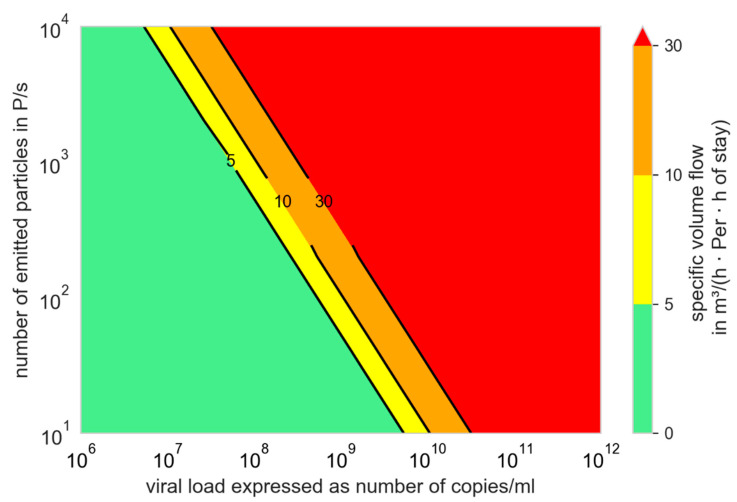
Specific volume flow depending on the number of emitted, particles and the viral load to limit the number of newly infected persons to one; N_0_ = 67 viral copies, f_M_ = 1, Q_b,in_ = Q_b,e_ = 0.54 m^3^/h.

If for a first assumption it is assumed that the ratio of change in the transmissibility is correlated with the ratio of change in the R value, which is furthermore inversely correlated with the critical dose, it can be assumed that an increase in the transmissibility of 50% ($\frac{N_{0}}{N}=\frac{1}{1.5}$) will result in a reduction in the critical dose from 100 viral copies to 67 viral copies. Figure 3 can therefore be converted into Figure 5 and Figure 8 into Figure 9.

### 3.3. Comparison of Prevention Measures: Ag Testing, Wearing Masks, and Increasing Ventilation Rate

As can be seen from Equation (35), the VF (S_v_/N_0_) is dominant in PAR_S_. S_v_/N_0_ varies by a factor of 1000 between a viral load of 10^8^ and 10^11^. The preventive measures (increasing the virus-free air supply volume, wearing masks, reducing the time of stay and their combination) in the specific situation have to be of a similar order of magnitude to actually prevent an outbreak. The comparison was performed with the simplified model, which is valid for lower PAR_s_, and in the case of high PAR_s_ further measures should be implemented, so that the actual value for higher PAR_s_ is not relevant. If the lower limit of the supplied virus-free air volume flow is calculated to reach a CO_2_ concentration of 4000 ppm (common for not regularly performed window ventilation and longer stays) and the volume flow is increased until a lowered CO_2_ concentration of 1000 ppm is reached (complies with the normative recommendation for indoor air quality), the factor of change is 7 related to the air volume flow and the preventive impact. Wearing a face mask, on average, reduces the inhaled dose by 50%, whereas a factor of 2 can also be applied for halving the time of stay in the room together with the infectious person. For a FFP2 mask the dose will, on average, be reduced by 80%, whereas a factor of 5 can be applied.

**Figure 10:**
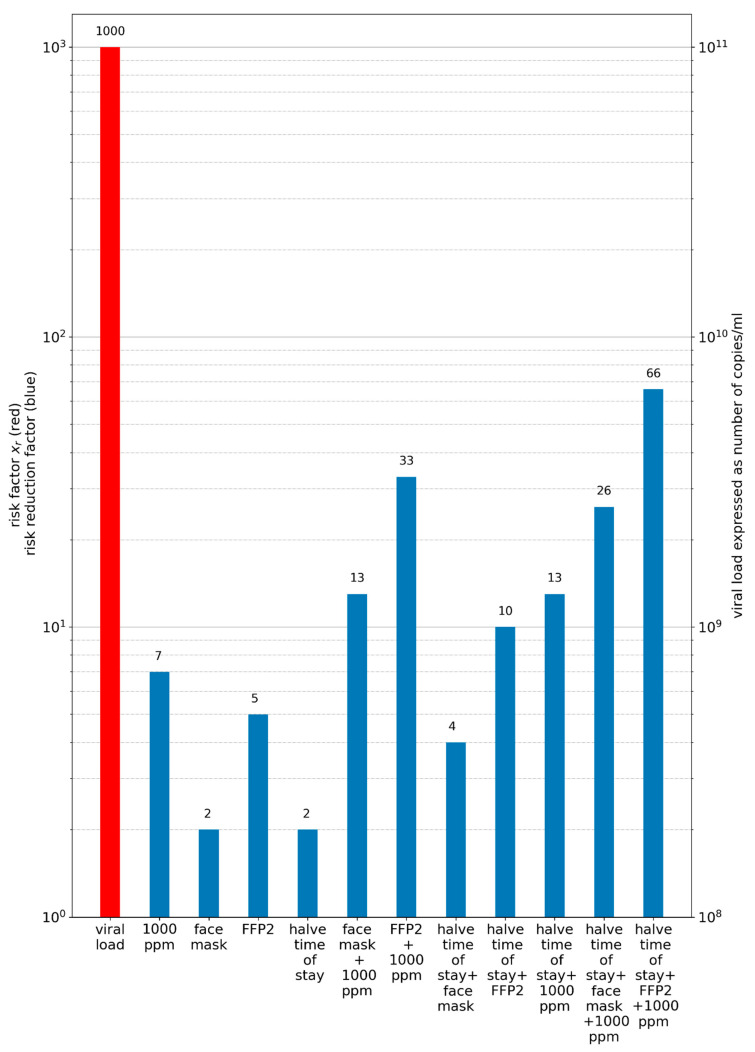
Influence of different preventive measures on the risk of an outbreak. The red bar represents the viral load and the resulting risk factor (Equation (37)). The blue bars illustrate different combinations of preventive measures in the form of a risk reduction factor (also according to Equation (37)).

From Figure 10, it can be seen that even with a combination of different measures an outbreak cannot be avoided completely, and infections may occur, if the viral load is high enough. A small change in viral load by a factor of 10, e.g., from 10^8^ to 10^9^, could probably be compensated for by wearing masks and ventilating regularly (Figure 10, blue bar: face mask + 1000 ppm). If the virus source entering the room can be avoided, it is obvious that this is the most effective preventive measure. Ag tests can be of practical use, even if their sensitivity is limited for low and medium viral load.

## 4. Discussion

A dose–response model to evaluate the risk of infection with SARS-CoV-2 was used to analyze twenty-five outbreaks in different situations (e.g., school, choir, meetings).

Although the viral loads in the investigated outbreaks were unknown, our results strongly suggest that relevant transmission will take place when viral loads are high. Data regarding particle emission rate during various activities were used to infer virus emission rate, but this varied significantly among individuals (Figure 2). Even if the particle emission is significantly under- or overestimated, a viral load of at least 108 viral copies/mL would have to exist (see Figure 3 and Figure 4). Using this particle emission rate and size distribution, the number of aerosolized viruses is obtained as a function of viral load. It is assumed that the viruses at the site of aerosol generation in the body correspond to the viral load in the swab, which remains to be proven.

The highest calculated value was found for a choir rehearsal with $\frac{S_{v}}{N_{0}}=2529\frac{1}{h}$ and is therefore within the range found by Buonanno et al. [32]. The lowest value of $\frac{S_{v}}{N_{0}}=36\frac{1}{h}$ is found for bus travel, where the activity and/or the viral load might have been low, which is also comparable to the results of Buonanno et al. [32]. The high emission during the choir rehearsal seems valid, because of the high particle emission rates while singing and the low air change rate. Furthermore, it has to be taken into consideration that the particle emission for different persons might be different for the same activity, so the $\frac{S_{v}}{N_{0}}$ can be quite different, but the main conclusion will remain.

Regardless of the correct quantity of particle emission strength and viral load, it was shown that the specific person-related airflow rate is a practical quantity for evaluating ventilation-related measures, simply mathematically derived; see (24)–(32).

It was also clearly demonstrated by us that preventive ventilation measures can prevent outbreaks due to aerosol transmission only in a narrow band. At high virus loads, the outbreak size can be reduced, but not prevented. Additional preventive measures are often necessary (Figure 5, Figure 9 and Figure 10).

If the viral load must be so high to cause infection due to aerosol transmission, then even using Ag tests can be a very effective measure to prevent infectious individuals from entering the room and causing an outbreak. Nevertheless, their efficiency depends on many influencing factors, such as the quality (sensitivity) of the test and the correct execution of the test. Even if studies showed that in general, most pupils are able to perform the Ag tests correctly if instructed, repetition on a regular basis may result in less attention paid to correct testing. Additionally, if antigen tests are not carried out on a daily basis, the possibility exists that a person with a positive test result already exhibited transmissible virus loads the previous day.

However, different limitations regarding the model for practical application have to be considered. First of all, different influencing factors (e.g., critical dose, decay rate of sedimentation and inactivation, size distribution and number of emitted particles) were assumed based on current knowledge, but whereas the transmission of SARS-CoV-2 is still ongoing, further knowledge may be gained from further research. The decay rates of sedimentation as well as inactivation can be influenced by the particle size distribution, as well as the air temperature and humidity in the room. Furthermore, the analysis and model are based on some general assumptions, such as the ideal mixing of all particles within the room, an initial concentration of 0 virus copies/m^3^ and a supply of virus-free air. An ideal mixing of all particles in the room also implies that no separation into the near and far field can be performed, whereas the concentration of virus-laden particles near the person is probably higher than in the rest of the room. Additionally, the local concentration will differ regularly from the average concentration, such that the local air quality index should be considered for investigation in detail [28,29]. As a result, even at lower viral emission rate, S_v_, infection can occur via aerosol, predominantly in the near field. As a third aspect, the influence of VOCs is difficult to define. A higher transmission rate of new VOCs may result from different aspects, such as a change in critical dose, a change in viral load or a change in other measures. It also has to be kept in mind that the investigated outbreaks documented with ARs from 4% to 100% had mostly high ARs and therefore resulted in a high number of newly infected persons. Many transmission events have much lower ARs, so the results may over- or understate the true risk.

It could be shown that for viral loads smaller than 10^8^ viral copies/mL, aerosol transmission becomes unlikely if the distance is maintained. However, it has to be considered that in some of the investigated cases, the range between the 25% and the 75% percentile is quite high, which is because of insecure boundary conditions.

## 5. Conclusions

(1) For an outbreak due to aerosol transmission to happen, high viral loads are required, which regularly occurs with the Delta variant.
(2) Preventive measures such as wearing masks and rising ventilation cannot prevent an outbreak when virus loads are very high, but are useful to mitigate it.
(3) The person-related air flow rate per hour of stay is a favorable indicator for evaluating the preventive effect of ventilation measures. According to our observations, even volume flow rate and person-related volume flow rate have a more informative quality than the air change rate.
(4) Instead of CO_2_ concentration, the CO_2_ dose (integration of the difference from the outdoor air concentration) is suitable for defining a limit value that should not be exceeded.
(5) With a simplified approach it is easy to compare different indoor situations and preventive measures regarding aerosol transmission.
(6) Ag tests possess an effective additional quality: they have a high sensitivity (detection rate) at virus loads of more than 10^6^ viral copies/mL and are therefore able to detect infectious persons, providing the chance to isolate them before entering a room for a longer stay.

From the investigation of the outbreaks, it can be concluded that in all cases a viral load of at least 10^8^ (viral copies)/mL was necessary to reach the observed attack rates. This demonstrates that the viral load estimated from the swab might overestimate a person’s infectivity via aerosol, because a person is generally considered infectious, independent of the transmission method, when the viral load from the swab is 10^6^ viral copies/mL. It can be seen that the viral emission of the infected person is the dominant influencing factor, but three further aspects (situational aspects, personal aspects of the susceptible persons and preventive measures) have to be considered to transfer a transmission of SARS-CoV-2 into a superspreading event. It can be concluded that higher activity (such as singing or physical activity) alone does not necessarily result in high ARs (Choir Rehearsal Berlin 2) and that lower activity may also result in high ARs (e.g., School Hamburg 1) if other unfavorable conditions (e.g., high viral load, ineffective preventive measures) occur.

Nevertheless, a higher activity will result in a higher emission rate of particles and therefore in a higher concentration of virus-laden particles in the room air. For transmission, aerosol production and viral load must always be considered together. A person being considered infectious does not mean that aerosol transmission will also occur.

The comparison of different preventive measures demonstrates that these measures alone as well as in combination are able to reduce the rate of transmission, but for high viral loads high infection rates will still occur. Especially with the VOC Delta, for which an up to 1000-times higher viral load was found, there is a high risk of outbreaks and superspreading events even when the AHA+L rules are observed (German abbreviation for masks, hand hygiene, social distancing and ventilation).

We showed that to recommend an air change rate $\lambda_{\mathrm{ACH}}$ alone is not sufficient for infection prevention, whereas the person-related volume flow of a virus-free air supply is a much more relevant parameter. The specific volume flow per person can be correlated with the CO_2_ concentration in the room, but whereas the number of inhaled virus-laden particles increases with time, the CO_2_ concentration will reach a steady-state concentration after a certain time, and does not change much until the persons leave the room. In comparison, the number of inhaled virus-laden particles increases over time even if their concentration in the room stays constant. Therefore, to use a fixed CO_2_ concentration as an indicator for the risk of infection has important limitations. Instead, the CO_2_ dose (ppm·h) can be used meaningfully and is easy to integrate in an infection risk monitoring system.

In the case of a high number of susceptible persons ($N_{\mathrm{pers}}>5$) and low predicted attack rates (${\mathrm{PAR}}_{s}<20\%$), a simplified model was set up, which can be used to predict the influence of different measures on the risk of infection, to calculate the maximum number of persons or to calculate the necessary volume flow per person to avoid the infection of more than one person, and is applicable to compare different indoor situations. Therefore, for high ARs the simplified model is not applicable; it is suitable to be used before outbreaks happen, where low ARs are expected. For retrospective analysis, a more detailed model should be used.

High viral loads, which may result in transmission via aerosol particles, can be found with antigen rapid tests. In this case, the exposition does not occur (if the person does not enter the room at all) or is of short duration (if the test is performed in the room).

Due to the fact that the concentration of viral copies in the surroundings of an infected person is always higher and that there are insecurities regarding the tests, as well as the transmission from or to vaccinated or recovered persons, a multilayer approach of preventative measures such as wearing masks and increasing the air flow rate are necessary to lower the infection rate.

For future outbreaks, it would be helpful if all boundary conditions (e.g., volume flow, room size, time of exposure, etc.) and the viral load at the moment of transmission were determined retrospectively. A summary of the necessary information for the boundary conditions is presented in Appendix B.

## Figures and Tables

**Table 1:** Results for the different factors for the investigated outbreaks.

	AR in %	Situation-Related Factor (SF)		$\mathrm{Susceptible}-\mathrm{Person}-\mathrm{Related}\;\mathrm{Factor}\;(\mathrm{SPF})\;Q_{b,\mathrm{in}}\;\mathrm{in}\;\frac{m3}{h}$	Personal-Protection-Measures-Related Factor (SPF) f_M_	Virus-Related Factor (VF)		$\frac{S_{v}}{N_{0}}$ $\mathrm{in}\;\frac{1}{h}(\mathrm{Monte}-\mathrm{Carlo}-\mathrm{Simulation})$		
		$C_{R}$ $\mathrm{in}\;\frac{h2}{m3}$	$C_{R,steady}$ $\mathrm{in}\;\frac{h2}{m3}$			$\frac{S_{v}}{N_{0}}$ $\mathrm{in}\;\frac{1}{h}$	${\frac{S_{v}}{N_{0}}}_{steady}$ $\mathrm{in}\;\frac{1}{h}$	Median	25% Percentile	75% Percentile
Choir Rehearsal Berlin 1 (A)	89	0.0013	0.0022	0.65	1	2529	1576	2594	2145	3220
Choir Rehearsal Berlin 2 (B)	24	0.006	0.0009	0.65	1	732	464	774	656	916
Skagit Valley Choir (C)	87	0.0019	0.0030	0.65	1	1649	1065	1932	1226	3335
French Choir (D)	68	0.0088	0.0165	0.65	1	199	107	200	144	281
Korean Call Center (E)	12	0.0018	0.0018	0.54	1	135	133	345	254	462
Korean Fitness Center (F)	30	0.0011	0.0019	0.9	1	378	205	375	283	495
Hawaiian Fitness Class (G)	100	0.0033	0.0098	0.9	1	2312	787	1014	523	1686
German Slaughterhouse (H)	26	0.0018	0.0020	0.9	1	185	167	184	150	226
School Israel (I)	43	0.0052	0.0058	0.54	1	216	195	140	103	184
Courtroom (J)	33	0.0115	0.0154	0.54	1	58	41	57	46	73
Wuhan Restaurant (K)	45	0.0096	0.0192	0.54	1	115	58	120	97	149
Aircraft (L)	62	0.0084	0.0084	0.54	1	214	213	212	173	261
Buddhist Bus (M)	34	0.0076	0.0090	0.54	1	102	86	99	72	133
Wuhan (Bus 1) (N)	15	0.0084	0.0094	0.54	1	36	32	35	25	48
Wuhan (Bus 2) (O)	17	0.0058	0.0079	0.54	1	59	44	59	45	78
Minivan 1 (P)	63	0.0124	0.0131	0.54	0.5	481	455	475	309	690
Minivan 2 (Q)	45	0.0124	0.0131	0.54	0.7	85	81	83	54	121
Club Meeting (R)	58	0.0029	0.0059	0.54	1	564	271	568	485	670
School Berlin 1 (S)	10	0.0038	0.0039	0.45	1	56	54	87	59	118
School Berlin 2 (T)	6	0.0015	0.0016	0.45	1	85	76	157	109	212
Meeting Germany (U)	17	0.0045	0.0060	0.54	1	77	58	79	64	97
School Hamburg 1 (V)	57	0.0062	0.0071	0.45	1	271	238	295	225	381
School Hamburg 2 (W)	33	0.0020	0.0024	0.45	1	401	334	456	344	592
School Hamburg 3 (X)	13	0.0020	0.0024	0.45	0.7	199	166	224	165	300
School Hamburg 4 (Y)	4	0.0008	0.0012	0.45	0.7	143	97	161	123	210
Min	4	0.0006	0.0009	-	-	36	32	35	25	48
Max	89	0.0160	0.0012	-	-	2529	1576	2594	2145	3320

AR—attack rate. $C_{R}$—situation-related factor (SF) considering the air change rate, deposition, inactivation, room volume and time of stay. $C_{R,\mathrm{steady}}$—situation-related factor in a steady situation considering the air change rate, deposition, inactivation, room volume and time of stay. Q_b,in_—inhalation flow rate of the susceptible persons. f_M_—mask efficiency considering the inhalation and exhalation efficiency. $\frac{S_{v}}{N_{0}}$—virus emission rate of the infectious person divided by the critical dose. ${\frac{S_{v}}{N_{0}}}_{\mathrm{steady}}$—virus emission rate of the infectious person divided by the critical dose in a steady situation.

## Appendix A

### Calculation of the Conversion Factor from Number to Volume of the Particles

In different studies, the particle emission rate for different activities was measured. The results found by some of the authors of this paper can be seen in Table A1. It has to be mentioned that the particles were measured about 0.81 m behind the mouth of the subjects. Therefore, it was assumed that they had already reached equilibrium diameter when entering the particle counter. To take this into consideration, the evaporation has to be considered. Regarding [42], it is considered that the equilibrium diameter is between 33 and 50% of the original diameter.

**Table A1:** Particle emission rates measured by the authors, partially published for adults in [34,35,39], for adolescents in [38] and for children in [40].

			Particle Emission Rate in Particles/s				
			Cumulative	0.3–0.5 µm	0.5–1.0 µm	1.0–3.0 µm	>3.0 µm
Adults [35,35,39]	Breathing through the nose (26 subjects)	Average	32	17	9	5	0
		Median	16	12	4	2	0
		Min	0	0	0	0	0
		Max	740	314	262	159	12
	Breathing through the mouth (37 subjects)	Average	164	87	48	27	1
		Median	68	36	16	10	0
		Min	2	0	0	0	0
		Max	1036	612	381	148	18
	Speaking (45 subjects)	Average	268	152	77	38	1
		Median	212	118	49	24	0
		Min	17	4	0	0	0
		Max	1194	730	330	275	25
	Coughing * (7 subjects)	Average	13,708	8047	3478	2057	126
		Median	9790	6494	2806	2315	98
		Min	1805	1099	392	314	0
		Max	287,697	196,781	71,826	18,933	353
	Singing (39 subjects)	Average	1511	842	458	208	2
		Median	1376	742	396	166	0
		Min	133	72	31	28	0
		Max	6215	3677	1989	1457	23
	Shouting (15 subjects)	Average	1843	1105	507	231	0
		Median	1295	777	353	165	0
		Min	330	141	94	71	0
		Max	5862	3743	1719	471	0
Adolescents (13–15 years) [38]	Breathing (7 subjects)	Average	54	18	16	19	1
		Median	41	17	5	14	0
		Min	0	0	0	0	0
		Max	749	352	201	179	17
	Speaking (8 subjects)	Average	112	65	25	20	3
		Median	98	51	20	11	1
		Min	13	5	5	0	0
		Max	251	137	55	53	13
	Singing (8 subjects)	Average	577	284	166	122	5
		Median	490	286	127	72	3
		Min	165	64	33	19	0
		Max	1229	529	337	345	17
	Shouting (8 subjects)	Average	2940	1491	820	603	26
		Median	2477	1417	697	461	9
		Min	720	410	151	160	0
		Max	5048	2486	1464	1088	104
Children (8–10 years) [40]	Breathing (15 subjects)	Average	2	2	0	0	0
		Median	0	0	0	0	0
		Min	0	0	0	0	0
		Max	23	23	0	0	0
	Speaking (15 subjects)	Average	40	27	3	8	2
		Median	24	24	0	0	0
		Min	0	0	0	0	0
		Max	118	71	47	47	23
	Singing (14 subjects)	Average	131	72	34	25	0
		Median	118	59	35	23	0
		Min	0	0	0	0	0
		Max	800	400	235	165	0
	Shouting (15 subjects)	Average	1166	614	298	250	5
		Median	1012	589	259	188	0
		Min	23	23	0	0	0
		Max	2260	1177	659	659	47

* Emission rate for 0coughing per cough.

To calculate the conversion factor from the particle emission rate to the volume of the particles, first of all, the volume of a particle has to be calculated. Spherical particles were assumed for this calculation. Furthermore, for each size class the minimum diameter is assumed to be the most representative, because more smaller than larger particles were found. With this assumption, the volume of the particles in the different size classes can be calculated with a shrinking factor of 2, so a particle with a diameter of 0.3 µm was calculated with an initial diameter of 0.6 µm.

$$V_{0.3}=1.13\cdot 10^{-13}\mathrm{mL}$$

$$V_{0.5}=5.24\cdot 10^{-13}\mathrm{mL}$$

$$V_{1.0}=4.19\cdot 10^{-12}\mathrm{mL}$$

$$V_{3.0}=1.13\cdot 10^{-10}\mathrm{mL}$$

For adults, the conversion factor is calculated with the following distribution of size classes when speaking: 57% 0.3–0.5 µm, 23% 0.5–1.0 µm, 18% 1.0–3.0 µm and 2% larger than 3.0 µm.

The average emission of volume by exhaling one particle can therefore be calculated using Equation (A1), where i denotes the size class, f_i_ the proportion of particles in the size class and V_i_ the volume of particles in this size class.

(A1) $$V_{\mathrm{exh},P}=\overset{4}{\underset{i=1}{\sum }}f_{i}\cdot V_{i}$$

For breathing, the exhaled volume of particles is therefore $V_{\mathrm{exh},P}=3.20\cdot 10^{-12}\frac{\mathrm{mL}}{P}$. The conversion factor shall further include the conversion from seconds to hours and is therefore $f_{p}=1.1523\cdot 10^{-8}\frac{\mathrm{mL}\cdot s}{P\cdot h}$.

## Appendix B

### Necessary Boundary Conditions to Retrospectively Investigate Outbreaks

For the investigation of outbreaks, different boundary conditions are necessary. The bold are elementary, the others optional. They are summarized in the following:

(1) Virus-related aspects
  - How many people were infected by the virus?
  - How many people attended the event?
  - How many people were vaccinated or had recovered from an infection?
  - Has it been defined which type of virus caused the infection? If yes, which one?
  - How high was the virus load during the infection event?
(2) Room-related aspects
  - How big is the room in which the event took place (area, volume)?
  - Was the room ventilated mechanically?
    - What volume flow or air change rate was available?
  - Was the room ventilated by window opening?
    - How often and for how long were the windows opened?
    - Is there anything known about the outdoor conditions on that day? (Temperature, wind speed, wind direction.)
(3) Event-related aspects
  - How long did the event take?
  - Did all attendees stay in the room together for the whole event? Otherwise, specify which part left and for how long.
  - What was the main activity of the infectious person?
  - What was the main activity of the susceptible persons?
  - Were there any additional preventive measures (e.g., face masks)?
  - Were the persons at fixed positions during the stay? What were the approximate positions of the persons?

## Appendix C

For twenty-five different outbreaks, information was available or was assumed.

Four different choir rehearsals were considered. All choirs rehearsed in unventilated or just slightly ventilated rooms. The number of attending persons ranged from 25 to 77 persons and the room size from 135 to 1720 m^3^. The duration of the rehearsals differed just slightly, between 2 and 2.5 h.

In the Choir Rehearsal Berlin 2, the previously infected person was the choir director and was therefore not singing the whole time. Therefore, a mixture of singing and speaking was assumed [40].

In the French Choir, three members of the choir tested positive for SARS-CoV-2 within the next few days after the rehearsal [74]. Although one member had symptom onset the day after the rehearsal, it seems reasonable to assume that only one person emitted the virus-laden particles.

The second group consists of outbreaks either involving loud speaking or intense physical activity such as sports or heavy working. For three out of the four outbreaks, little information regarding the ventilation system in the room is available, so typical values were assumed, either by the authors of this study (Hawaiian Fitness Class [75]) or by the authors of earlier studies (Korean Call Center and Korean Fitness Center [31]). Solely for the German Slaughterhouse, measurements of the volume flow were performed afterwards [76].

For the working places (call center [77] and slaughterhouse [76]) the time of stay was a working day (8 h), whereas for the fitness class the duration of stay was significantly smaller (approximately 1 h or less [75,78].

Two outbreaks with normal office activity, but relatively high attack rates are considered as well [79,80]. One outbreak happened during a school day (4.5 h) in a ventilated classroom [79] and the other during a court session (3 h) in an unventilated courtroom [80]. Both rooms had a similar size. Furthermore, an outbreak in a restaurant was included in this category. It is one of the first documented outbreaks related to the airborne transmission of SARS-CoV-2. The boundary conditions, mainly room volume and ventilation, were assumed by Li et al. [1].

In addition, some outbreaks related to public transport were found [81,82,83,84]. One outbreak happened in an aircraft during a 11 h flight with 217 passengers and the other in buses of different sizes (3 to 68 passengers and 16 to 71 m^3^). The bus travel lasted between 1 and 2.5 h.

Finally, two meetings and six outbreaks in school classes, which were investigated and provided by local health authorities or the Robert Koch Institute, are considered. For the outbreaks, the air change rate was either assumed afterwards depending on the ventilation habits and the temperature and wind speed on the day of outbreak or known from existing ventilation systems. The room volume, the number of attending persons and the time of stay were investigated by the health authorities together with the attack rate reported for this outbreak.

The index person in two of the school cases (School Hamburg 3 and School Hamburg 4) wore a mask (f_M_ = 0.7) and noted some symptoms, whereas he reduced speaking intensity.

**Table A2:** Scientifically published SARS-CoV-2 outbreaks.

	Choir Rehearsal Berlin 1	Choir Rehearsal Berlin 2	Skagit Valley Choir	French Choir	Korean Call Center	Korean Fitness Center	Hawaiian Fitness Class	German Slaughterhouse
Source/s	[40]	[40]	[30,31]	[74]	[31,77]	[31,78]	[75]	[76]
Air change rate in 1/h	0.17	0.45	[31] 1.5, [30] 0.3–1.0	unventilated, assumed 0.1	[31] 1.5	[31] 1.5	unventilated, assumed 0.1	0.53
	0 ± 50%	0 ± 50%	0 ± 50%	0 ± 50%	0 ± 50%	0 ± 50%	0 ± 50%	++ ±5%
Room volume in m^3^	1200	1720	810	135	1143 **	180	114 ***	3000
	++ ±5%	++ ±5%	+ ±20%	+ ±20%	+ ±20%	+ ±20%	0 ± 50%	+ ±20%
Number of previously infected persons	1	1	1	1 *	1	1	1	1
	++ ±5%	++ ±5%	++ ±5%	0 ± 50%	++ ±5%	++ ±5%	++ ±5%	++ ±5%
Number of susceptible persons	77	42	61	25	89	192	10	78
Main activity	Singing	Singing/Speaking	Singing	Singing	Speaking	Physical Activity	Physical Activity	Heavy working
Breathing volume flow (Q_b,e_ and Q_b,in_) in m^3^/h	0.65	0.61	0.65	0.65	0.54	0.9	0.9	0.9
	+ ±20%	+ ±20%	+ ±20%	+ ±20%	+ ±20%	+ ±20%	+ ±20%	+ ±20%
Mask efficiency in %	1	1	1	1	1	1	1	1
	-	-	-	-	-	-	-	-
Time of stay in h	2.5	2	2.5	2	8	0.8	1	8
	++ ±5%	++ ±5%	++ ±5%	++ ±5%	+ ±20%	++ ±5%	++ ±5%	+ ±20%
Attack rate in %	89	24	87	68	65	30	100	26
	++ ±5%	++ ±5%	+ ±20%	+ ±20%	++ ±5%	+ ±20%	+ ±20%	++ ±5%

Grey background color—Evaluation of the security of the boundary conditions ++ quite secure, + somewhat insecure, 0 unknown/insecure, assumed level of deviation. * Three persons had contact with a person who later tested positive, but just one person had symptom onset on the day after the rehearsal. ** Transmission occurred in a volume of 600 m^3^. *** Area given, height of 3 m assumed.

**Table A3:** Scientifically published SARS-CoV-2 outbreaks.

	School Israel	Courtroom	Wuhan Restaurant	Aircraft	Buddhist Bus	Wuhan (Bus 1)	Wuhan (Bus 2)	Minivan 1	Minivan 2
Source/s	[79]	[80]	[1,85]	[81]	[31,82]	[31,83]	[31,83]	[84]	[84]
Air change rate in 1/h	2.7	unknown, assumed 0.3	[1] 0.56–0.77	21	3	3	3	9	9
	0 ± 50%	0 ± 50%	0 ± 50%	+ ±20%	0 ± 50%	0 ± 50%	0 ± 50%	0 ± 50%	0 ± 50%
Room volume in m^3^	150	150	[19] 431 *	60 **	50	71	34	16	16
	++ ±5%	++ ±5%	+ ±20%	++ ±5%	+ ±20%	+ ±20%	+ ±20%	+ ±20%	+ ±20%
Number of previously infected persons	1	1	1	1	1	1	1	1	1
	++ ±5%	++ ±5%	++ ±5%	++ ±5%	++ ±5%	++ ±5%	++ ±5%	++ ±5%	++ ±5%
Number of susceptible persons	66	9	21	19	68	48	12, 1 with mask	4, all with cloth masks	3, infected person w/o mask, other persons w/mask
Main activity	Speaking	Speaking	Speaking	Speaking	Speaking	Speaking	Speaking	Speaking	Speaking
Breathing volume flow (Q_b,e_ and Q_b,in_) in m^3^/h	0.5	0.54	0.54	0.54	0.54	0.54	0.54	0.54	0.54
	+ ±20%	+ ±20%	+ ±20%	+ ±20%	+ ±20%	+ ±20%	+ ±20%	+ ±20%	+ ±20%
Mask efficiency in %	1	1	1	1	1	1	1	0.7	0.85
	-	-	-	-	-	-	-	+ ±20%	+ ±20%
Time of stay in h	4.5	3	1.2	11	1.7	2.5	1.0	2	2
	++ ±5%	++ ±5%	++ ±5%	++ ±5%	++ ±5%	++ ±5%	++ ±5%	++ ±5%	++ ±5%
Attack Rate in %	43	33	45	6	34	15	17	100	33
	++ ±5%	+ ±20%	++ ±5%	++ ±5%	++ ±5%	++ ±5%	++ ±5%	++ ±5%	++ ±5%

Grey background color—Evaluation of the security of the boundary conditions ++ quite secure, + somewhat insecure, 0 unknown/insecure, assumed level of deviation. * Transmission occurred in a volume of 47 m^3^. ** Only the Business Class is included where the majority of infections occurred. Evaluation of the security of the boundary conditions ++ quite secure, + somewhat insecure, 0 unknown/insecure, assumed level of deviation.

**Table A4:** Small outbreaks investigated by the Robert Koch Institute or local health authorities.

	Club Meeting	School Berlin 1	School Berlin 2	Meeting Germany	School Hamburg 1	School Hamburg 2	School Hamburg 3	School Hamburg 4
Source/s	[86]	[86]	[86]	[86]	[87]	[87]	[87]	[87]
Air change rate in 1/h	0.20	8.3	10	1.2	1.9	3.3	3.2	3.2
	0 ± 50%	0 ± 50%	0 ± 50%	0 ± 50%	0 ± 50%	0 ± 50%	0 ± 50%	0 ± 50%
Room volume in m^3^	254	180	150	170	154	157	157	157
	++ ±5%	++ ±5%	++ ±5%	++ ±5%	++ ±5%	++ ±5%	++ ±5%	++ ±5%
Number of previously infected persons	1	1	1	1	1	1	1	1
	++ ±5%	++ ±5%	++ ±5%	++ ±5%	++ ±5%	++ ±5%	++ ±5%	++ ±5%
Number of susceptible persons	25	27	20	11	28	24	24	27
Main activity	Speaking	Speaking	Speaking	Speaking	Speaking	Speaking	Speaking	Speaking
Breathing volume flow (Q_b,e_ and Q_b,in_) in m^3^/h	0.54	0.45	0.45	0.54	0.54/0.45 *	0.54/0.45 *	0.54/0.45 *	0.54/0.45 *
	+ ±20%	+ ±20%	+ ±20%	+ ±20%	+ ±20%	+ ±20%	+ ±20%	+ ±20%
Mask efficiency in %	1	1	1	1	1	1	0.7	0.7
	-	-	-	-	-	-	+ ±20%	+ ±20%
Time of stay in h	1.5	4.5	1.5	2	3	1.5	1.5	0.75
	++ ±5%	++ ±5%	++ ±5%	++ ±5%	++ ±5%	++ ±5%	++ ±5%	++ ±5%
Attack rate in %	58	10	6	17	57	33	13	4
	++ ±5%	++ ±5%	++ ±5%	++ ±5%	++ ±5%	++ ±5%	++ ±5%	++ ±5%

Grey background color—Evaluation of the security of the boundary conditions ++ quite secure, + somewhat insecure, 0 unknown/insecure, assumed level of deviation. * Exhalation/inhalation breathing volume flow.

## Appendix D

### Comparison of Different Situations

To compare different situations with the simplified approach, a viral load of 10^10^ viral copies/mL was used. For the situation in the supermarket with mask, which is currently common, R_s_ = 1 is used as the reference. In all situations, the duration of stay is longer than in the supermarket, but in the theater/cinema scenario the risk is lower than in the supermarket. In all other situations the risk is higher, and if the duration of stay is much longer (office or school) the risk is significantly higher and further measures (reduction in the number of persons in the room, wearing masks) are necessary to reduce the risk.

**Table A5:** Comparison of different situations with the simplified approach.

	N_p_ in P/s	S_v_/N_0_ in Viral Copies/h	t in h	q_pers_ in m^3^/h×Per	Q_b,in_ in m^3^/h	f_M_ in -	R_s_ in Per	x_r_ in -
Reference: supermarket, with mask	160	184	0.5	25	0.54	0.5	1	1
Office half occupancy, without mask	160	184	8	60	0.54	1	13.2	13.2
Office half occupancy, with mask	160	184	8	60	0.54	0.5	6.6	6.6
School normal occupancy, without mask	80	92	6	25	0.54	1	11.9	11.9
School half occupancy, without mask	80	92	6	50	0.54	1	6	6
School half occupancy, with mask	80	92	6	50	0.54	0.5	3	3
Restaurant normal occupancy	160	184	1.5	50	0.54	1	6	6
Restaurant half occupancy	160	184	1.5	100	0.54	1	3	3
Theater/cinema half occupancy, with mask	80	92	2	60	0.54	0.5	0.8	0.8

Grey background—reference case. N_p_—particle emission rate. $\frac{S_{v}}{N_{0}}$—virus emission rate of the infectious person divided by the critical dose. t, time of stay. q_pers_—specific volume flow per person. Q_b,in_—inhalation flow rate of the susceptible persons. f_M_—mask efficiency considering the inhalation and exhalation efficiency. R_s_—number of newly infected persons in a specific situation. x_r_—risk factor for a specific situation.
